# Rare Case of Bilateral and Asymmetrical Multiple Evanescent White Dot Syndrome

**DOI:** 10.7759/cureus.37937

**Published:** 2023-04-21

**Authors:** Kumutha Muthusamy, Norshamsiah Md Din, Nor Fadzillah Bt Abd Jalil

**Affiliations:** 1 Ophthalmology, Pusat Perubatan Universiti Kebangsaan Malaysia (PPUKM), Kuala Lumpur, MYS; 2 Ophthalmology, Hospital Melaka, Melaka, MYS; 3 Ophthalmology, Universiti Kebangsaan Malaysia Medical Centre, Kuala Lumpur, MYS

**Keywords:** intraretinal punctate lesions, complete resolution, sd-oct, asymmetrical, bilateral multiple evanescent white dot syndrome

## Abstract

Bilateral presentation of multiple evanescent white dot syndrome (MEWDS) is a rare occurrence. We report a case of bilateral multiple evanescent white dot syndrome in a young female patient with asymmetrical manifestation. She presented with sudden onset of right eye central blurring of vision and dyschromatopsia. Fundus examination however showed bilateral multiple grey-white intra-retinal punctate lesions with an asymmetrical manifestation of the swollen optic disc and foveal granularity over the right. Spectral Domain Optical Coherence Tomography (SD-OCT) showed the presence of juxta foveal subretinal fluid and disrupted inner segment-outer segment (IS-OS) junction over the right eye. The patient had a spontaneous complete recovery within six weeks’ time.

## Introduction

Multiple evanescent white dot syndrome (MEWDS) belongs to a spectrum of diseases known as the white dot syndromes. It commonly affects healthy young myopic females where 1/3rd of the cases occur following a viral prodrome [1]. This condition is typically unilateral whereby patients present with numerous whitish dots seen in the posterior pole and the mid periphery which is limited in time and may not be present when the patient consults [2]. The pathophysiology of MEWDS is still unknown although recent studies show that it could be caused by inflammatory choriocapillaries non-perfusion leading to ischemia of the outer retina and damage to the outer segment of photoreceptors [3]. We would hereby report a rare case of bilateral and asymmetrical manifestation of MEWDS in a young female patient in our setting.

## Case presentation

**Figure 1:**
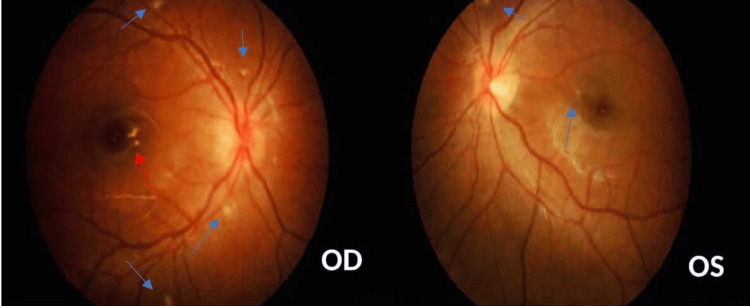
Fundus photograph showing the presence of multiple grey-white subretinal punctate lesions (blue arrows) bilaterally with the presence of swollen optic disc and foveal granularity (red arrow) over the right eye

A 17-year-old girl presented with sudden onset of right eye central blurring of vision associated with dyschromatopsia for one week. She has denied any preceding viral prodrome and there were also no symptoms suggestive of tuberculosis. She is myopic and has been wearing spectacles for the past five years. The best-corrected vision at the presentation was 6/18 OD and 6/9 OS. There was no relative afferent pupillary defect. Bilateral anterior segment examination was unremarkable. Fundus examination of the bilateral eye however revealed multiple grey-white sub-retinal punctate lesions with an asymmetrical manifestation of the swollen optic disc and foveal granularity over the right (Figure 1). Otherwise, there was no associated vitritis, vasculitis, or retinitis bilaterally.

**Figure 2:**
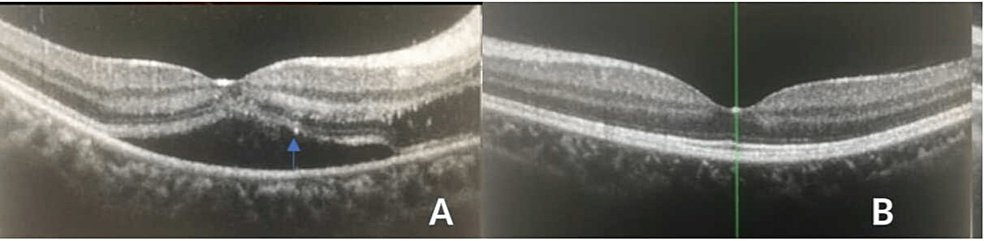
A: SD-OCT macula of the right eye showed juxta-foveal subretinal fluid with IS/OS junction disruption (arrow). B: Normal SD-OCT macula of the left eye Spectral Domain Optical Coherence Tomography (SD-OCT), Inner Segment and Outer Segment (IS/OS)

The optic nerve function test revealed impaired light brightness and red saturation over the right eye. The neurological examination was otherwise unremarkable. Humphrey Visual Field (HVF) test showed a slightly enlarged blind spot over the right eye and was normal over the left. Spectral Domain - Optical Coherence Tomography (SD-OCT) of the macula showed the presence of juxta-foveal subretinal fluid with disrupted inner segment-outer segment (IS-OS) junction over the right eye (Figure 2A) and was normal over the left (Figure 2B).

**Figure 3:**
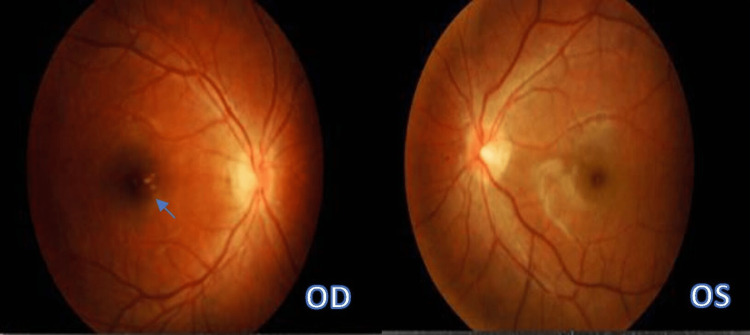
Spontaneous resolution of intraretinal lesions and optic disc swelling after six weeks seen bilaterally with the persistence of foveal granularity over the right eye.

Fundus Fluorescein Angiography (FFA) and Indocyanine Green Angiography (ICGA) were unable to be carried out for the patient as it was not available in our facility and the patient was not keen on external referral in view of travel restrictions following the Covid pandemic at the time of diagnosis. Blood investigations including Full Blood Count, Erythrocyte Sedimentation Rate (ESR), Rapid Plasma Reagent (RPR), and Anti-Nuclear Antibody (ANA) showed normal findings. Her chest x-ray was normal and the Mantoux test was insignificant. Based on history, clinical findings, and investigation, the patient was diagnosed to have bilateral Multiple Evanescent White Dot Syndrome (MEWDS). She was followed up with a serial fundus photo and SD-OCT macula scans. Her condition resolved spontaneously after six weeks and her best-corrected vision upon her last visit was 6/9 bilaterally. There was complete resolution of the intraretinal punctate lesions however foveal granularity persisted over the right eye (Figure 3).

**Figure 4:**
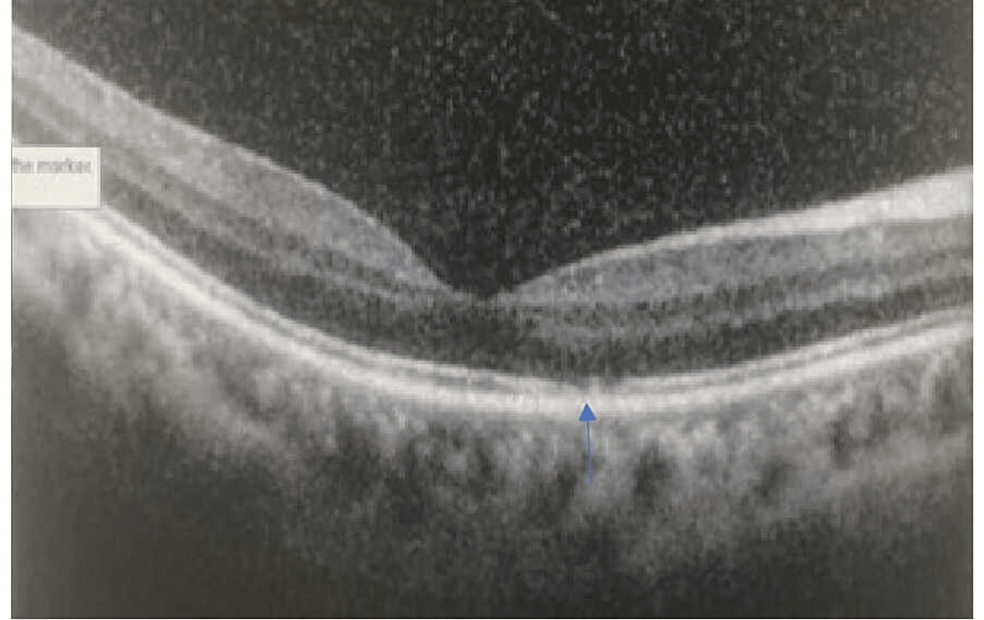
SD-OCT macula of the right eye show resolution of subretinal fluid after six weeks however IS/OS junction disruption persists (blue arrow). Spectral - Domain Optical Coherence Tomography (SD-OCT), Inner Segment and Outer Segment (IS/OS)

Serial SD-OCT macula scan over the right eye showed resolution of juxtafoveal subretinal fluid as well however disruption of IS/OS junction still persisted after six weeks (Figure 4).

## Discussion

Multiple evanescent white dot syndrome (MEWDS) could be a challenging diagnosis as many ocular conditions have a similar presentation. In this patient, the diagnosis was made based on suggestive clinical history, examination, and imaging findings however bilaterality is uncommon in MEWDS, and other sight and life-threatening conditions such as infection, masquerade, and inflammatory disorder need to be ruled out. Conditions that often have MEWDS-like presentation are such as syphilis, tuberculosis, primary intraocular lymphoma, sarcoidosis, and other white dot syndromes such as acute posterior multifocal placoid pigment epitheliopathy (APMPPE) and acute zonal occult outer retinopathy (AZOOR) [4]. Bilateral MEWDS has been infrequently reported whereby there have so far only six cases have been reported in the literature and all of them had asymmetric presentations as seen in this patient [5].

Foveal granularity is one of the characteristic and distinguishing features of MEWDS where it is seen in 74-96% of cases, and it could be the only presenting sign in the post-acute phase due to the transient nature of the white dots [6]. Macula edema is one of the contributing factors for poor vision in patients with MEWDS as it is also seen in this patient. Retinal pigment epithelium (RPE) dysfunction in MEWDS could have caused the macula edema which will disappear spontaneously with the improvement of the RPE dysfunction over time [7]. On the other hand, peri papillary nerve fiber swelling is a common finding which may be secondary to the retrograde spread of RPE photoreceptor inflammation to the optic nerve [8].

Multimodal imaging plays an important role in the diagnosis of bilateral and asymmetric MEWDS as the less affected eye could be asymptomatic at presentation [9]. Spectral Domain Optical Coherent Tomography (SD-OCT) is a useful non-invasive imaging for diagnosis and monitoring as there would be disruption of the photoreceptor IS-OS junction seen in the affected eye [10]. Indocyanine green angiography (ICGA), fundus autofluorescence (FAF), and fundus fluorescein angiography (FFA) aid in diagnosis in complement with the clinical findings. FFA reveals “wreath-like” early hyperfluorescent spots with late staining whereby IGGA findings consist of patchy hyperfluorescent areas in the posterior pole, mid periphery as well as around the optic disc. Fundus autofluorescence will show bright autofluorescent patterns that become iso-fluorescent after photobleaching with blue light.

MEWDS is a benign self-limiting condition that resolves spontaneously. Recurrences and immunosuppressive treatment are only seen in less than 10% of the cases [11]. The recovery of visual function is often seen within several weeks as is observed in this patient as well. Foveal granularity however may persist for a longer duration, but it is not associated with poor visual recovery. Focal choroidal excavation and choroidal neovascularization (CNV) are rare complications of MEWDS and may appear months or years after the resolution of the disease [12].

## Conclusions

MEWDS is a benign condition that may mimic many other conditions and shall be made as a diagnosis of exclusion. Bilateral presentation is a rarity hence thorough history taking, physical examination and multimodal imaging techniques will be useful in clinical practice. A high index of suspicion must be maintained in patients who do not achieve any spontaneous resolution.
